# Brazilian nursing specific situation, middle and micro-range theories: a bibliometric study

**DOI:** 10.1590/0034-7167-2023-0520

**Published:** 2024-09-20

**Authors:** Rosane Barreto Cardoso, Marcos Antônio Gomes Brandão, Jéssica Cristina Sobrinho da Silva Cavalcante, Rafael Oliveira Pitta Lopes, Kênia Rocha Leite Zaccaro, Cândida Caniçali Primo

**Affiliations:** I Universidade Federal do Rio de Janeiro. Rio de Janeiro, Rio de Janeiro, Brazil; II Universidade Federal do Espírito Santo. Vitória, Espírito Santo, Brazil

**Keywords:** Nursing Theory, Nursing Models, Health Postgraduate Programs, Nursing Research, Bibliometrics, Teoría de Enfermería, Modelos de Enfermería, Programas de Posgrado en Salud, Investigación en Enfermería, Bibliometría

## Abstract

**Objectives:**

to map the nursing theories developed in stricto sensu graduate programs in nursing in Brazil.

**Methods:**

a bibliometric study, carried out on the Coordination for the Improvement of Higher Education Personnel Theses and Dissertations Portal in October 2023. The controlled descriptors “Nursing Theory” and “Nursing Models” and the uncontrolled descriptors “Theories” and “Middle-Range Theory” were used. Selected studies were cataloged for analysis, which was conducted by the study authors, who have a doctoral degree and expertise in research on nursing theories.

**Results:**

thirty-nine nursing theories were mapped, with a predominance of middle-range theories (79.5%), focusing on nursing diagnostic concepts and use of the theoretical-methodology strategy of theoretical-causal validity.

**Conclusions:**

the study identified nursing theories developed in Brazil, recognizing trends, development strategies, theorized objects of disciplinary interest and investments necessary for practical application in Brazilian contexts.

## RESUMEN

**Objetivos:**

mapear las teorías de enfermería desarrolladas en los programas de posgrado de enfermería stricto sensu en Brasil.

**Métodos:**

estudio bibliométrico, realizado en el Portal de Tesis y Disertaciones de la Coordinación para el Perfeccionamiento del Personal de Educación Superior en octubre de 2023. Los descriptores controlados “Teoría de Enfermería” y “Modelos de Enfermería” y los descriptores no controlados “Teorías” y “Rango Medio Teoría”. Los estudios seleccionados fueron catalogados para el análisis, que fue realizado por los autores del estudio, quienes tienen doctorado y experiencia en investigación sobre teorías de enfermería.

**Resultados:**

se mapearon 39 teorías de enfermería, con predominio del desarrollo de teorías de mediano alcance (79,5%), con foco en conceptos diagnósticos de enfermería y utilización de la estrategia teórico-metodológica de validez teórico-causal.

**Conclusiones:**

el estudio identificó teorías de enfermería desarrolladas en Brasil, reconociendo tendencias, estrategias de desarrollo, objetos teorizados de interés disciplinar e inversiones necesarias para su aplicación práctica en contextos brasileños.

## INTRODUCTION

For decades, theorists and metatheorists have argued that nursing theories are relevant to knowledge and practice, producing something that could be recognized as a Nursing Theory Guide Practice (NTGP) paradigm. The theories have contributed to a unique knowledge base necessary for better understanding human phenomena and responses^(1-2)^.

In 2020, Ahtisham Younas questioned the preponderance of NTGP by applying Imre Lakatos’ philosophical criteria. It was pointed out a probable NTGP degeneration as a scientific research program, when compared to evidence-based practice and knowledge translation^(3)^. The argument for NTGP degeneration was supported by findings from an integrative literature review in English to examine the use of theories to guide experimental research and compare their usefulness with traditional nursing practice^(3)^, and from a review of 61 doctoral theses in nursing science produced in English between 1994-2015 at the University of Edinburgh, in the United Kingdom^(4)^.

On the other hand, there are studies that still indicate the current use of theories as a reference framework for research and practices and nursing theory development in English, particularly^(5-6)^ which would weaken the degeneration argument.

Brazil accounts for the majority of nursing production in Portuguese, and has worldwide relevance in training master’s and doctoral professionals in the area as well as a tradition in the academic defense of NTGP. Studies covering the period from 1998 to 2019 demonstrate that, in Brazil, studies that used nursing theories are based mainly on North American theorists; however, they do not indicate the advancement or degeneration of the theorizing movement in Brazilian nursing^(7-9)^.

Brazilian authors published a literature review^(10)^ based on articles to describe how middle-range theories (MRTs) for nursing were developed until 2016. Of the 25 studies found, none were published in Portuguese, and only one had Brazilian authors in its list^(10)^. However, there were already MRTs for nursing produced in the country and deposited in university repositories and in the Coordination for the Improvement of Higher Education Personnel (CAPES - *Coordenação de Aperfeiçoamento de Pessoal de Nível Superior*) Journal Portal in the form of theses, which were not identified in the article.

Evidence indicates a gap in current knowledge about nursing theory development in Brazil. There is a growing interest in nursing theory development, especially middle-range ones^(11)^. Thus, our research question is: what is the overview of nursing theory production in Brazil according to dissertation and theses reports from graduate programs in nursing?

Graduate programs at universities are the main environments for nursing theory production in Brazil. Therefore, it is essential to look for dissertation and theses reports to understand the real state of knowledge about theories developed in Brazil.

## OBJECTIVES

To map nursing theories developed in *stricto sensu* graduate programs in nursing in Brazil.

## METHODS

### Ethical aspects

The research was not submitted for consideration by the Research Ethics Committee, since it was carried out with secondary data in the public domain. However, study copyright was preserved.

### Study design, period and place

This is a bibliometric study carried out based on the analysis of dissertations and theses from Brazilian graduate programs in nursing deposited in the CAPES Theses and Dissertations Catalog. This type of work was chosen because it represents the high level of scientific production in the country, presenting methodological rigor in studies, validated by qualification boards.

The studies were located using the CAPES Theses and Dissertations Catalog search tool, using the Health Sciences Descriptors (DeCS): “Nursing Theory” and “Nursing Models”. The terms “Theory” and “Middle-Range Theory” were also applied, and the Preferred Reporting Items for Systematic Reviews and Meta-Analyses (PRISMA) was implemented. There was no time frame delimitation. Data collection took place in October 2023.

### Inclusion and exclusion criteria

Dissertations and theses that developed nursing theories without time span were included. Research that was not available online was excluded.

### Study protocol

Data search and extraction were carried out by two researchers who were authors of the study. Any disagreements were discussed among all authors.

**Figure 1:**
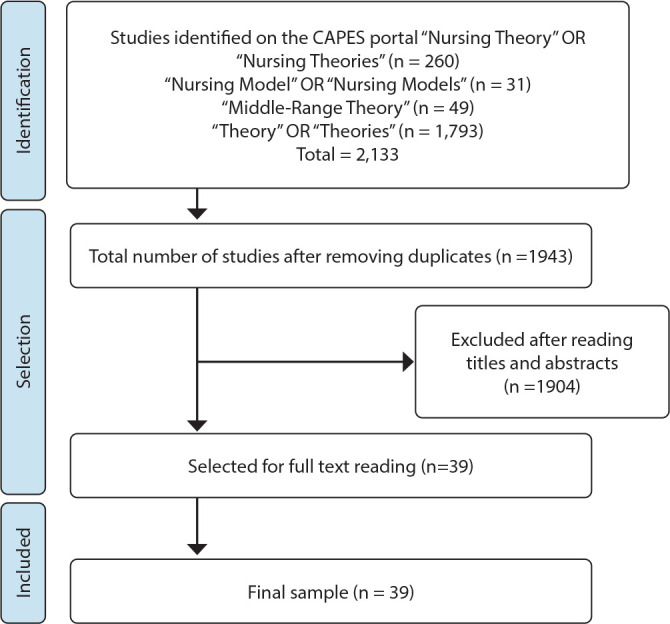
Study selection flowchart adapted from PRISMA, Rio de Janeiro, Rio de Janeiro, Brazil, 2023

The following strategies were applied in the search field of the CAPES Theses and Dissertations Catalog: “Nursing Theory” (descriptor) OR “Nursing Theories” (keyword), with 260 studies found; “Nursing Models” (descriptor) OR “Nursing Model” (keyword), with 31 studies found; “Mid-Range Theory” (keyword), with 49 studies found; and “Theory” OR “Theories” (keywords), with 1,793 studies found. The filters “health sciences” from the “major area of knowledge” and “nursing”/“public health nursing”/“obstetric nursing”/“pediatric nursing” from the “area of knowledge” were used. After reading the titles and abstracts, 1,904 studies were excluded because they did not address theory development. The final sample consisted of 39 studies (Figure 1).

### Analysis of results, and statistics

The data were recorded in an electronic spreadsheet that included information on the year of completion, study title, keywords, study objective, university, author, advisor, program, name of theory/model developed, study design, theoretical/methodological framework and area of knowledge established by the authors (women’s health, adult/elderly health, child/adolescent health and mental health).

The cataloged data were analyzed by the study authors, who have doctoral degrees and expertise in research on nursing theories, in addition to academic studies in this area.

Descriptive statistics with absolute and relative frequency were used to analyze the data, and the findings were presented in charts/tables.

## RESULTS

Thirty-nine nursing theories were found, produced and disseminated in 26 (66.7%) theses and 13 (33.3%) dissertations, from 2012 to 2023, in 13 graduate programs in three geographic regions of Brazil. Chart 1 presents a summary of the profile of theories (T), organized in ascending order of year, informing the title of thesis or dissertation, institution, authors, advisors and main objectives.

**Chart 1:** Distribution of theses and dissertations produced in Brazil about nursing theory development (n=39), Rio de Janeiro, Rio de Janeiro, Brazil, 2023

Number year university	Thesis/dissertation Title	Author Advisor	Main objectives
(T1) 2012 UFRJ	Thesis: *Proposição de uma Teoria de Enfermagem para o Processo de Interação em Ambientes Virtuais* ^(12)^	Author: Jaqueline Santos de Andrade Martins Advisor: Marcos Antônio Gomes Brandão	Propose a nursing theory for the process of interaction in virtual environments based on the theory-building strategies proposed by Walker and Avant.
(T2) 2015 UFRJ	Thesis: *Teoria de Médio Alcance de Amamentação: tecnologia de cuidado* ^(13)^	Author: Cândida Caniçali Primo Advisor: Marcos Antônio Gomes Brandão	Develop an MRT of breastfeeding based on Imogene King’s Conceptual Model of Open Systems.
(T3) 2016 UFRJ	Thesis: *Teoria de Médio Alcance de Enfermagem para Atenção à Saúde Mental* ^(14)^	Author: Wilson Denadai Advisor: Marcos Antônio Gomes Brandão	Propose an MRT for nursing for mental health based on Imogene King and the conceptual and philosophical aspects of the Brazilian Health System.
(T4) 2017 UFC	Dissertation: *Padrão respiratório ineficaz em crianças com cardiopatias congênitas: construção e validação por juízes de uma Teoria de Médio Alcance* ^(15)^	Author: Nayara Maria Gomes de Souza Advisor: Viviane Martins da Silva	Verify the validity of the structure of the nursing diagnosis (ND) Ineffective Breathing Pattern in children with congenital heart disease by carrying out two stages, such as MRT construction and analysis by judges.
(T5) 2018 UECE	Dissertation: *Reabilitação cardiovascular: proposta de uma teoria de enfermagem de médio alcance* ^(16)^	Author: Maria Sinara Farias Advisor: Lucia de Fatima da Silva	Develop an MRT for nursing based on theory-building strategies proposed by Walker and Avant for the process of cardiovascular rehabilitation.
(T6) 2018 UECE	Thesis: *Teoria de enfermagem de médio alcance para adesão de pessoas ao tratamento de hipertensão arterial e diabetes mellitus* ^(17)^	Author: Kalidia Felipe de Lima Costa Advisor: Maria Vilani Cavalcante Guedes	Develop and validate a nursing MRT for adherence of individuals to treatment for high blood pressure and diabetes mellitus.
(T7) 2018 UFRN	Thesis: *Construção e validação do diagnóstico de enfermagem risco de volume de líquidos excessivo a partir de uma Teoria de Médio Alcance* ^(18)^	Author: Maria Isabel da Conceição Dias Fernandes Advisor: Ana Luísa Brandão de Carvalho Lira	Construct and validate the ND Risk for Excess Fluid Volume in patients undergoing hemodialysis based on an MRT.
(T8) 2019 UFSC	Dissertation: *Modelo Teórico de Cuidado de Enfermagem às Mulheres em Tratamento de Câncer de Mama na Saúde Suplementar* ^(19)^	Author: Ana Carolina Custódio Advisor: Evangelia Kotzias Atherino dos Santos	Develop a theoretical model of nursing care for women undergoing breast cancer treatment in light of complex thinking in the supplementary health sector.
(T9) 2019 UFSM	Thesis: *Estressores e variances de bem-estar em pessoas idosas hospitalizadas: Teoria de Médio Alcance de enfermagem* ^(20)^	Author: Eliane Raquel Rieth Benetti Advisor: Margrid Beuter	Develop a nursing MRT on stressors and variances in well-being in hospitalized older adults based on the theoretical synthesis of empirical data and the derivation of concepts from the Neuman Systems Model.
(T10) 2019 UFPB	Thesis: *Subconjunto terminológico da CIPE^®^ para pessoas com síndrome metabólica: base conceitual para a Teoria de Médio Alcance do cuidado no contexto de risco cardiovascular* ^(21)^	Author: Nuno Damácio de Carvalho Félix Advisor: Maria Miriam Lima da Nóbrega	Analyze the concepts of cardiovascular risk and metabolic syndrome; structure a terminological subset of ICNP^®^; perform the clinical application of subset; and develop an MRT for care in the context of cardiovascular risk.
(T11) 2019 UFC	Thesis: *Validação do diagnóstico de enfermagem resposta disfuncional ao desmame ventilatório (RDDV)* ^(22)^	Author: Larissa de Araújo Lemos Advisor: Marcos Venício de Oliveira Lopes	Establish the validity of the ND Dysfunctional Ventilatory Weaning Response from NANDA-I and develop an MRT on dysfunctional ventilatory weaning.
(T12) 2019 UFPE	Dissertation: *Validação do conteúdo do diagnóstico de enfermagem “conhecimento deficiente em indivíduos com insuficiência cardíaca”* ^(23)^	Author: Claudia Gabrielle da Silva Advisor: Cecília Maria Farias de Queiroz Frazão	Validate the content of the ND Deficient Knowledge in individuals with heart failure and build an MRT with the identification of attributes, antecedents and consequences.
(T13) 2020 UFRJ	Thesis: *Teoria do risco de padrão glicêmico desequilibrado em adultos e idosos com diabetes mellitus em tratamento* ^(24)^	Author: Rafael Oliveira Pitta Lopes Advisor: Marcos Antônio Gomes Brandão	Develop a theory on glycemic variations in adults and older adults with diabetes mellitus undergoing treatment, based on Roy’s Conceptual Adaptation Model.
(T14) 2020 UFC	Thesis: *Teoria de situação específica e análise de conteúdo do diagnóstico de enfermagem nutrição desequilibrada: menor do que as necessidades corporais em crianças com câncer* ^(25)^	Author: Iane Ximenes Teixeira Advisor: Marcos Venício de Oliveira Lopes	Develop two stages of the ND validity process in order to obtain evidence of theoretical and content validity of the ND Imbalanced Nutrition: Less than Body Requirements in the context of children with cancer.
(T15) 2020 UFRN	Thesis: *Ressecamento Ocular: desenvolvimento de uma teoria de enfermagem de médio alcance* ^(26)^	Author: Ana Paula Nunes de Lima Fernandes Advisor: Allyne Fortes Vitor	Develop an MRT for nursing on dry eye based on Roy’s Adaptation Model.
(T16) 2020 UFPE	Thesis: *Validação do diagnóstico de enfermagem rede social de apoio ineficaz* ^(27)^	Author: Michelline Santos De Franca Advisor: Cleide Maria Pontes	Validate the ND Inadequate Social Support Network and develop the MRT Inadequate Social Support Network.
(T17) 2020 UFC	Thesis: *Validação do diagnóstico de enfermagem proteção ineficaz em adolescentes com câncer* ^(28)^	Author: Marilia Mendes Nunes Advisor: Marcos Venício de Oliveira Lopes	Establish a diagnostic structure that represents evidence of validity for inference of the ND Ineffective Protection in adolescents with cancer and construct an MRT.
(T18) 2021 UFRN	Dissertation: *Construção de uma teoria de médio alcance para o diagnóstico de enfermagem estilo de vida sedentário* ^(29)^	Author: Renata Marinho Fernandes Advisor: Ana Luísa Brandão de Carvalho Lira	Construct an MRT for the ND Sedentary Lifestyle in adolescents and young adults.
(T19) 2021 UFPR	Thesis: *Teoria de enfermagem de médio alcance para o cuidado transpessoal domiciliar* ^(30)^	Author: Luana Tonin Advisor: Maria Ribeiro Lacerda	Construct an MRT for nursing for transpersonal home care.
(T20) 2021 UFRN	Dissertation: *Sobrepeso em adolescentes e adultos jovens: uma teoria de médio alcance* ^(31)^	Author: Ana Carolina Costa Carino Advisor: Ana Luísa Brandão de Carvalho Lira	Construct an MRT for the ND Overweight in adolescents and young adults.
(T21) 2021 UFRN	Thesis: *Teoria de médio alcance para o cuidado de enfermagem ao adolescente com obesidade na atenção primária à saúde* ^(32)^	Author: Anne Karoline Candido Araújo Advisor: Ana Luísa Brandão de Carvalho Lira	Develop an MRT for nursing care in Primary Health Care for adolescents with obesity.
(T22) 2021 UFSM	Thesis: *Cuidado centrado à pessoa idosa institucionalizada com demência: teoria de médio alcance de enfermagem* ^(33)^	Author: Larissa Venturini Advisor: Margrid Beuter	Develop an MRT for nursing care for institutionalized older adults with dementia, based on Carl Rogers’ Person-Centered Therapy.
(T23) 2021 UFMA	Dissertation: *Análise de conteúdo do diagnóstico de enfermagem perfusão tissular periférica ineficaz em pacientes com pé diabético* ^(34)^	Author: Lorrany Fontenele Moraes Da Silva Advisor: Livia Maia Pascoal	Analyze the content of the ND Ineffective Peripheral Tissue Perfusion in patients with diabetic foot based on an MRT.
(T24) 2022 UFRN	Dissertation: *Teoria de médio alcance do diagnóstico de enfermagem de risco de aspiração em pacientes críticos adultos* ^(35)^	Author: Priscila Kaline de Andrade Silva Rocha Advisor: Ana Luísa Brandão de Carvalho Lira	Develop an MRT for the ND Risk for Aspiration in critical patients.
(T25) 2022 UFSM	Thesis: *Microteoria para cuidados de enfermagem na prevenção do delirium em pessoas idosas na unidade de terapia intensiva* ^(36)^	Author: Sandra da Silva Kinalsk Advisor Margrid Beuter	Develop a prescriptive microtheory for nursing care in delirium prevention in older adults in the Intensive Care Unit based on constructs from Callista Roy’s Adaptation Model.
(T26) 2022 UFRN	Thesis: *Desenvolvimento de teoria de médio alcance de enfermagem para letramento em saúde prejudicado* ^(37)^	Author: Amanda Barbosa da Silva Advisor: Allyne Fortes Vitor	Develop an MRT for impaired health literacy.
(T27) 2022 UFPB	Thesis: *Aplicabilidade clínica do subconjunto terminológico da CIPE^®^ para a mulher idosa com vulnerabilidade relacionada ao HIV/AIDS: desenvolvimento de uma teoria de médio alcance* ^(38)^	Author: Marcia Cristina de Figueiredo Santos Advisor: Maria Miriam Lima da Nóbrega	Assess the clinical applicability of the ICNP^®^ terminology subset for elderly women with HIV/AIDS-related vulnerability, structuring an MRT.
(T28) 2022 UFRJ	Thesis: *Teoria de médio alcance do processo adaptativo da pessoa com lesão na medula espinhal baseado no modelo de adaptação de Roy* ^(39)^	Author: Kenia Rocha Leite Zaccaro Advisor: Marcos Antônio Gomes Brandão	Construct an MRT of the adaptive process of a person with traumatic spinal cord injury based on Callista Roy’s Adaptation Model.
(T29) 2022 UFRGS	Thesis: *Teoria de médio alcance da dimensão existencial do ser no mundo da doença renal crônica* ^(40)^	Author: Carolina Giordani da Silva Advisor: Maria da Graça Oliveira Crossetti	Develop an MRT of the existential dimension of being in the world of chronic kidney disease based on the Humanistic Nursing Theory.
(T30) 2022 UFF	Thesis: *Diagnóstico de enfermagem Letramento em Saúde Insuficiente na População Idosa: Estudo de validação* ^(41)^	Author: Rachel da Silva Serejo Cardoso Advisor: Rosimere Ferreira Santana	Assess evidence of conceptual, content and theoretical-causal validity of the diagnostic proposal Insufficient Health Literacy for older adults. Explanatory MRT based on the ND Insufficient Health Literacy in older adults.
(T31) 2022 UFRN	Dissertation: *Teoria de médio alcance para a Autogestão Ineficaz da Saúde no cuidado de pessoas vivendo com o Vírus da Imunodeficiência Humana* ^(42)^	Author: Rebecca Stefany Da Costa Santos Advisor: Richardson Augusto Rosendo da Silva	Develop an MRT for nursing for Ineffective Health Self-Management in people living with the Human Immunodeficiency Virus in light of Roy’s Adaptation Model.
(T32) 2022 UFC	Thesis: *Validação do diagnóstico de enfermagem baixa autoeficácia em saúde* ^(43)^	Author: Reinaldo Gutierrez Barreiro Advisor: Marcos Venício de Oliveira Lopes	Validate the diagnostic construct of Low Self-Efficacy in Health for possible incorporation into the NANDA International ND Classification and develop an MRT on Low Self-Efficacy in Health.
(T33) 2022 UFPE	Dissertation: *Validade de Conteúdo do diagnóstico de enfermagem Enfrentamento Ineficaz para Pacientes Oncológicos* ^(44)^	Author: Ingrid Andrade Lima Advisor: Eliane Maria Ribeiro de Vasconcelos	Investigate the content validity of the ND Ineffective Coping for cancer patients, constructed from an MRT.
(T34) 2022 UFC	Dissertation: *Validação de conteúdo do diagnóstico de enfermagem “síndrome pós-trauma” no contexto da violência contra a mulher* ^(45)^	Author: Yanka Alcantara Cavalcante Advisor: Marcos Venício de Oliveira Lopes	Verify the structural validity of the ND Post-Trauma Syndrome (PTS), through the production of two stages, such as construction of a situation-specific theory (SST), through the phases of an MRT, and content validity by judges.
(T35) 2023 UFRJ	Thesis: *Teoria do risco de quedas em pessoas idosas na comunidade* ^(46)^	Author: Jéssica de Castro Santos Advisor: Marcos Antônio Gomes Brandão	Construct a nursing theory on the risk of falls in older adults in the community.
(T36) 2023 UFRN	Thesis: *Desenvolvimento e Validação de Conteúdo da Proposição do diagnóstico de enfermagem Estresse Ocupacional a partir de uma teoria de médio alcance* ^(47)^	Author: Romanniny Hevillyn Silva Costa Almino Advisor: Richardson Augusto Rosendo da Silva	Develop and validate the content of the ND Occupational Stress proposal.
(T37) 2023 UFRN	Dissertation: *Desenvolvimento de uma teoria de médio alcance de Enfermagem para a Tensão do papel de cuidado* ^(48)^	Author: Barbara Ebilizarda Coutinho Advisor: Allyne Fortes Vitor	Develop an MRT for nursing for Caregiver Role Strain.
(T38) 2023 UNIFESP	Thesis: *Teoria de situação-específica para o controle da saúde na insuficiência cardíaca* ^(49)^	Author: Gisele Saraiva Bispo Hirano Advisor: Alba Lúcia Bottura L. de Barros	Construct an SST that defines and describes the health management of outpatients with heart failure.
(T39) 2023 UFRN	Dissertation: *Desenvolvimento de teoria de médio alcance de enfermagem para comportamentos ineficazes de manutenção da saúde em pessoas com condições crônicas* ^(50)^	Author: Ana Clara Dantas Advisor: Allyne Fortes Vitor	Develop an MRT for nursing for Ineffective Health Maintenance Behaviors in people with chronic conditions.

Table 1 shows the region of Brazil where the graduate program of the authors of the theory is located and details the higher education institutions.

**Table 1:** Characterization of dissertations and theses according to the regions of Brazil and higher education institution (n=39), Rio de Janeiro, Rio de Janeiro, Brazil, 2023

Region	Higher education institution	Dissertation n	Thesis n	Total f (%)
Northeast	*Universidade Federal do Rio Grande do Norte* (UFRN)	6	5	11(28.2)
	*Universidade Estadual do Ceará* (UECE)	1	1	2 (5.1)
	*Universidade Federal do Ceará* (UFC)	2	4	6 (15.4)
	*Universidade Federal da Paraíba* (UFPB)	0	2	2 (5.1)
	*Universidade Federal do Pernambuco* (UFPE)	2	1	3 (7.7)
	*Universidade Federal do Maranhão* (UFMA)	1	0	1 (2.6)
Southeast	*Universidade Federal do Rio de Janeiro* (UFRJ)	0	6	6 (15.4)
	*Universidade Federal de São Paulo* (UNIFESP)	0	1	1 (2.6)
	*Universidade Federal Fluminense* (UFF)	0	1	1 (2.6)
South	*Universidade Federal de Santa Catarina* (UFSC)	1	0	1 (2.6)
	*Universidade Federal de Santa Maria* (UFSM)	0	3	3 (7.7)
	*Universidade Federal do Paraná* (UFPR)	0	1	1 (2.6)
	*Universidade Federal do Rio de Janeiro* (UFRGS)	0	1	1 (2.6)

Theories produced in graduate programs in the Northeast region predominated (64.1%), followed by the Southeast (20.5%) and South (15.4%). The 39 theories (T) were developed by single authors and supervised by 18 researchers. The largest number of supervisions by the same advisor was six (T1, T2, T3, T13, T28, T35) from UFRJ, followed by five (T7, T18, T20, T21 and T24) from UFRN and five (T11, T14, T17, T32 and T34) from UFC. These advisors accounted for 41.0% of the supervisions.

**Figure 2:**
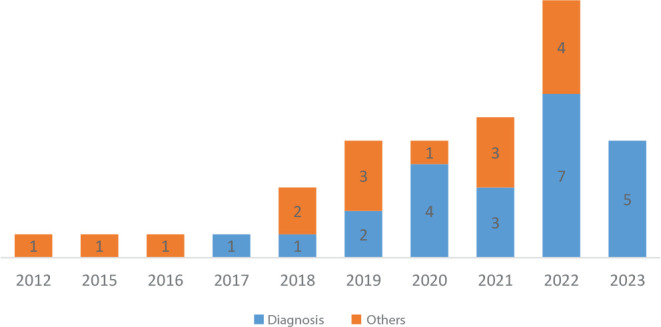
Evolutionary distribution of theories focusing on nursing diagnoses and other central conditions or concepts (n=39), Rio de Janeiro, Rio de Janeiro, Brazil, 2023

The predominant objectives of research were to create/develop nursing theories for ND validity, followed by clinical theorizations of conditions of interest to nursing, including for the proposition of prescriptive actions or connections with other elements of practice, such as nursing outcomes and interventions. Figure 2 establishes the temporal evolution between the generated/created theories of NDs and others.

Most theories focus on diagnostic concepts, mainly from the NANDA International (NANDA-I) classification, which is a growing trend over the years from 2020 onwards.

Regarding human groups or target populations, the theories include adults/older adults (71.8%; n=28), children/adolescents (12.8%; n=5), women (10.3%; n=4) and people with mental health disorders (2.6%; n=1). Only one theory (2.6%; n=1), the first identified (T1), was not directly related to clinical conditions, being focused on the process of interaction in virtual environments.

The authors classified their theories into the typologies of middle and micro, and SST, employing nine theorizing methods/strategies, as detailed in Chart 2.

**Chart 2:** Description of theorizing methods/strategies related to the type of theory developed (n=39), Rio de Janeiro, Rio de Janeiro, Brazil, 2023

Main theorizing methods/strategies used	MRT	SST	McT	NI
Analysis, synthesis and derivation of concepts, statements and theories^(51)^.	T1, T2, T6, T9, T10, T15, T19, T21, T22, T27	T38		
Theoretical-causal validity^(52)^.	T4, T5, T7, T11, T12, T16, T17, T18, T20, T23, T24, T26, T30, T31, T32, T33, T37, T39	T14, T34		
Use of explicit conceptual model to guide research^(53)^.	T36			
Grounded Theory^(54)^.	T29			T8
Dubin’s methodology for theory building^(55)^.				T13
Analysis of conceptual model, knowledge unpacking and theoretical combination^(56-57)^.	T3			
General method of theory development in applied disciplines^(58)^.				T35
Integrated classification-theory strategy^(2)^.	T28			
Theoretical substruction^(59-60)^.			T25	

*MRT - middle-range theory, SST - specific situation theory; McT - micro-range theory or microtheory; NI - not informed.*

The majority included MRT (82.1%; n=32), followed by SST (7.7%; n=3), micro-range theory or microtheory (McT) (2.6%; n=1), and in 7.7% (n=3) of the research the authors did not classify the theory.

Regarding the most prevalent strategies for developing theories, 51.3% used theoretical-causal validity from Lopes, Silva and Herdman; 28.2% used Walker and Avant’s strategies (analysis, synthesis and/or derivation of concepts, statements and theories); and 5.1% used Grounded Theory (GT).

## DISCUSSION

The mapping carried out provides evidence of a growing movement of development of nursing theories by master’s and doctoral students in Brazil, mainly from 2018 onwards, after the four^(12-15)^ pioneering studies verified from 2012 to 2017. This finding contradicts the idea of a degeneration in the nursing theory research program in the country, particularly for MRT for nursing.

It is difficult to assess the scope of the use of nursing theories as a framework for professional practice or in clinical nursing research, since it always requires the analysis of hundreds of studies that do not always clearly explain the theoretical frameworks. On the other hand, it is easier to map theoretical construction as a research enterprise. Such theories are basically created to constitute a basis for ND validity for the description, explanation or prediction of phenomena and conditions of interest for clinical practice or to prescriptively guide professional practice.

We also found that the movement that began in southeastern Brazil gradually spread to the northeast and then to the south. This spread is relevant for the expansion of theorizing, with the possibility of expanding capillarization and incorporating cultural and regional traits of a country of continental dimensions.

The predominance of theories developed in northeastern Brazil, accelerated from 2020 onwards, is associated with the relevant contribution of this region of the country in the development of research in ND theoretical-causal validity. The current criteria for the levels of evidence of NANDA-I NDs foresee the development of a theoretical foundation at the level of theoretical-causal validity^(52,61)^. The growth of graduate programs in Brazil, encouraged by the CAPES National Graduate Plan (PNPG 2011-2020), may also be related to this movement^(62)^.

We can interpret as an effort to theorize for clinical nursing practice the fact that 38 nursing theories address diagnoses or conditions of human groups or populations. Whether for groups of adults, older adults, children, women or groups with specific health problems, the theories communicate a direction for instrumentation of particular conditions of nursing care, moving away from more general themes or constructs, such as grand nursing theories. It also indicates less interest in theorizing the dimensions of teaching or management of professional practice^(9)^.

The limited focus also appears to be related to the predominant typologies of MRT, McT and SST. Such findings reinforce the perspective of levels of theoretical thinking, in which middle-range ones would have scope and abstraction between the specific to guide practice and research, however without losing the ability to assist different populations with the same phenomenon.

The predominance of MRTs, verified in mapping, converges with the trend of global theorization in nursing, which was observed from philosopher Frederick Suppe’s considerations on the structure of scientific theories and possibly from his presentation “Middle range theories: what they are and why nursing science needs them”, held in 1993, at the Council of Nurse Researchers Symposium of the American Nurses Association (ANA)^(63)^. Since then, there has been a continued interest in MRT construction to clarify, describe, prescribe and predict the phenomena, scenarios, facts, situations and interventions that are part of everyday nursing practice, contributing to the advancement of research and science in nursing^(64)^.

SSTs were the second most frequently verified typology in mapping, and they are also of low level of abstraction. In their original format, they followed more practical, contextualized and cultural traits compatible with the vocation of the University of California at San Francisco School of Nursing^(6)^. Among the philosophical roots that drive SSTs is post-empiricism, which aims to predict the experiences and responses of a given group of human beings under certain health and disease conditions^(65)^. However, currently, both the strategies for creating SSTs and the philosophical foundations are several^(65)^.

At the lowest levels of theoretical thinking, McTs are the least formal, restricted in time, scope and application, comprising groups of hypotheses of works or propositions^(63)^. This research identified the use of theoretical substructure as a strategy for creating the only microtheory^(36)^ identified.

Finally, the third type of theory at the lowest level of abstraction, which are practical theories, were not found in this review. Practical theories emerged in the 1960s based on the considerations of Dickoff *et al*.^(66)^ with a practice-oriented theorizing approach, with levels of factor isolation, factor relationship, situation relationship and situation production. Such theories would have the ultimate goal of modifying reality by prescribing situations, which depended on the preliminary stages of concept isolation with description, concept relationship and predictive statements^(6)^.

However, only recently, with the development of taxonomies and other types of theories, have theories of practice been able to advance to contemporary challenges, through the use of methods such as integrated classification-theory strategy^(2)^.

The predominance of strategies of synthesis, derivation and analysis of concepts, statements and theories was verified in non-diagnostic nursing theory elaboration. For Des’ theories, theoretical-causal validity strategy predominated.

In synthesis strategy, information based on observation is used to construct a new concept, a new statement, and a new theory. Derivation consists of transposing or redefining a concept, statement, and theory from one context or field to another. Meanwhile, in analysis, the theorist must dissect the whole into parts so that it can be better understood. Each of these strategies can be used to develop concepts, statements, and/or theories^(51)^.

The theoretical-causal validity strategy^(52)^ has driven the theoretical foundation for ND validity since 2017. This strategy, which is based on Roy’s model^(6)^, proposes theoretical development based on the following steps: definition of MRT construction approach; definition of theoretical-conceptual models to be analyzed; definition of MRT’s main concepts; development of a pictorial scheme; construction of MRT propositions; establishment of causality and evidence relationships for practice^(52)^.

Theorizing strategies that are not widely recognized in nursing were used more by theorists from southeastern and southern Brazil (T3, T8, T13, T25, T28, T29 and T35), indicating a tendency towards the use of new methods.

We can state, based on the research objectives and the elements produced in theorizing, that theorists were concerned with translation of knowledge, since, for the most part, they proposed some element of a more operational nature, such as diagrams, terminological subsets, action/intervention guides, links between diagnoses, ouctomes and interventions, among others. Although the classic elements of theoretical construction were present, such as concepts, premises, statements, propositions and iconic models, the authors predominantly produced explanatory, predictive or prescriptive elements in their theories, which brings an interesting approach to how much these theories could contribute to the purposes of practical theories and NTGP.

This aspect of theories approximated to practice conditions produces a counterpoint to the criticism of the relevance of nursing theories to the nursing profession. Perhaps the essence of this criticism is based on some clinicians’ perception about some of the grand North American nursing theories. The theory-practice gap has been described in the United States of America between 1980 and 1990, encompassing differences between the theories taught in the classroom and the practice found in the areas of clinical practice or even the existence of a disconnect between theoretical and practical realities^(67)^.

Thus, the progression of theoretical research verified in Brazil may be a factor that prevents or minimizes the NTGP program degeneration in Brazil, by offering new references that guide research and clinical practice. The mapped theories have a scope for clinical practice conditions or preparation for its exercise in challenging scenarios (virtual environments - T1).

Furthermore, the content of these theories may include regional aspects and the characteristics of the country’s health system. In fact, there must be an alignment between nursing theories and professional practice in order to achieve good and best practices, especially to collaborate in solving the challenges of the Brazilian Health System^(68)^.

### Study limitations

The study has some limitations. Full access to theses and dissertations depended on their location on the CAPES portal or in institutional repositories. Thus, theories that had not yet been deposited or were not in digital format may not have been included in mapping. To mitigate these limitations, a free search was conducted on the internet for the titles of selected dissertations and theses.

Another limitation was the use of a limited set of descriptors in the portal. Although we applied controlled descriptors and relevant uncontrolled terms, this restricted selection may result in loss of relevant studies, such as those related to theories produced by the GT method. It is worth noting that, although GT produces theories, its meaning of substantive and formal theories is usually not considered by researchers in this field as aligned with the most widespread idea of nursing theories. Given this little advanced debate, we chose to consider only theories in which the authors clearly announced a perspective convergent with the vision of nursing theories adopted in this study.

### Contributions to health and nursing

The research presented perspectives on scientific production of Brazilian nursing theories, highlighting the need to value and develop other studies, such as those validating and applying these theories.

Mapping enabled an understanding of the diversity and breadth of nursing theories developed in Brazilian academic contexts, offering insight into the current knowledge landscape in the field. Moreover, it provided valuable insights for improving graduate programs in nursing, allowing academic institutions to adjust their curricula and research approaches according to identified needs and trends. The study also helped identify areas in which nursing theories have not yet been widely explored or developed, highlighting potential gaps in knowledge that could be targets for future research.

## CONCLUSIONS

The bibliometric study made it possible to understand the nursing theories developed in graduate programs in Brazil, which represents an advance in the production of this knowledge in professional development and in the adoption of a practice program based on theories.

The advancement of nursing over the years is notable, with an increase in the production of theses and dissertations with research into nursing theory development. MRTs were the most prevalent in this bibliometrics and are continually growing, however, with studies still limited in the theoretical-methodological detail of its construction, which makes it difficult to consult theses and dissertations as educational material for developing theories. However, the use of these scientific studies in the country is still incipient, considering that theories support nursing science. Therefore, it is necessary to disseminate Brazilian nursing theories so that they can be incorporated into care practice (as support in nursing process implementation), teaching and research.
