# ERRATUM

**DOI:** 10.1590/0034-7167.20247706e08

**Published:** 2025-01-13

In the article “Brazilian nursing specific situation, middle and micro-range theories: a bibliometric study”, with DOI number: https://doi.org/10.1590/0034-7167-2023-0520, published in Revista Brasileira de Enfermagem, 2024;77(4):e20230520, Chart 1:

Where it read:

Author: Ingrid Andrade Lima

Advisor: Eliane Maria Ribeiro de Vasconcelos

Read:

Author: Ingrid Andrade Lima

Advisor: Jaqueline Galdino Albuquerque Perrelli

