# Supplementary material for: Brazilian nursing specific situation, middle and micro-range theories: a bibliometric study
Source: Rev Bras Enferm. 2024 Sep 20;77(4):e20230520. doi: 10.1590/0034-7167-2023-0520 (PMC11419682; doi:10.1590/0034-7167-2023-0520)
Supplement: Translation

# Translation: Teorias de enfermagem brasileiras de situação específica, médio e micro alcances: estudo bibliométrico

**DOI:** 10.1590/0034-7167-2023-0520pt

**Authors:** Rosane Barreto Cardoso, Marcos Antônio Gomes Brandão, Jéssica Cristina Sobrinho da Silva Cavalcante, Rafael Oliveira Pitta Lopes, Kênia Rocha Leite Zaccaro, Cândida Caniçali Primo

**Affiliations:** I Universidade Federal do Rio de Janeiro. Rio de Janeiro, Rio de Janeiro, Brasil; II Universidade Federal do Espírito Santo. Vitória, Espírito Santo, Brasil

**Keywords:** Teoria de Enfermagem, Modelos de Enfermagem, Programas de Pós-Graduação em Saúde, Pesquisa em Enfermagem, Bibliometria

## Abstract

**Objetivos:**

mapear as teorias de enfermagem desenvolvidas em programas de pós-graduação *stricto sensu* em enfermagem no Brasil.

**Métodos:**

estudo bibliométrico, realizado no Portal de Teses e Dissertações da Coordenação de Aperfeiçoamento de Pessoal de Nível Superior em outubro de 2023. Foram utilizados os descritores controlados “Teoria de Enfermagem” e “Modelos de Enfermagem” e os não controlados “Teorias” e “Teoria de Médio Alcance”. Os estudos selecionados foram catalogados para análise, que foi conduzida pelos autores do estudo, que possuem doutorado e *expertise* em pesquisas sobre teorias de enfermagem.

**Resultados:**

foram mapeadas 39 teorias de enfermagem, com predomínio de desenvolvimento de teorias de médio alcance (79,5%), com foco em conceitos diagnósticos de enfermagem e utilização da estratégia teórico-metodologia de validade teórico-causal.

**Conclusões:**

o estudo identificou as teorias de enfermagem desenvolvidas no Brasil, reconhecendo tendências, estratégias de desenvolvimento, objetos de interesse disciplinar teorizados e investimentos necessários para aplicação prática nos contextos brasileiros.

## INTRODUÇÃO

Por décadas, teoristas e metateoristas têm defendido que as teorias de enfermagem são relevantes ao conhecimento e à prática, produzindo algo que poderia ser reconhecido como um paradigma de prática guiada por teorias de enfermagem (*Nursing Theory Guide Practice* (NTGP), em inglês). As teorias contribuíram para uma base de conhecimento singular necessária ao melhor entendimento de fenômenos e respostas humanas^(1-2)^.

Em 2020, Ahtisham Younas pôs em questão a preponderância da NTGP aplicando os critérios filosóficos de Imre Lakatos. Apontou-se para uma provável degeneração da NTGP como programa de pesquisa científica, quando comparada à prática baseada em evidências e translação do conhecimento^(3)^. O argumento da degeneração da NTGP foi sustentado em achados de revisão integrativa de literatura, em inglês, para examinar o uso das teorias para guiar pesquisas experimentais e comparar a utilidade com a prática de enfermagem tradicional^(3)^, e de uma revisão de 61 teses de doutorado da ciência da enfermagem produzidas em inglês entre 1994-2015 na Universidade de Edinburgh, no Reino Unido^(4)^.

Por outro lado, existem publicações que ainda indicam o uso corrente de teorias como estrutura de referência das pesquisas e práticas e do desenvolvimento de teorias de enfermagem em língua inglesa, particularmente^(5-6)^ o que enfraqueceria o argumento de degeneração.

O Brasil é responsável pela maior parte da produção de enfermagem em língua portuguesa, e tem relevância mundial na formação de mestres e doutores na área, bem como uma tradição na defesa acadêmica da NTGP. Publicações cobrindo o período de 1998 a 2019 demonstram que, no Brasil, os estudos que usaram teorias de enfermagem se baseiam principalmente em teoristas norte-americanas; contudo, não indicam o avanço ou degeneração do movimento de teorização na enfermagem brasileira^(7-9)^.

Autores brasileiros publicaram revisão de literatura^(10)^ com base em artigos para descrever como foram desenvolvidas as teorias de médio alcance (TMAs) de enfermagem até 2016. Das 25 publicações encontradas, nenhuma fora publicada em português, e apenas uma possuía em seu elenco autores brasileiros^(10)^. Entretanto, já existiam TMAs de enfermagem produzidas no país e depositadas nos repositórios de universidades e no Portal de Periódicos da Coordenação de Aperfeiçoamento de Pessoal de Nível Superior (CAPES) em formato de teses, que não foram identificadas no artigo.

As evidências indicam uma lacuna no conhecimento atual sobre a elaboração de teorias de enfermagem no Brasil. Observa-se um crescente interesse no desenvolvimento de teorias de enfermagem, especialmente as de médio alcance^(11)^. Assim, temos como questão de pesquisa: qual é o panorama da produção de teorias de enfermagem no Brasil de acordo com os relatórios de dissertações e teses dos programas de pós-graduação em enfermagem?

Os programas de pós-graduação das universidades são os principais ambientes para a produção de teorias de enfermagem no Brasil. Assim, é essencial buscar os relatórios de dissertações e teses para conhecer o real estado do conhecimento sobre teorias desenvolvidas no Brasil.

## OBJETIVOS

Mapear as teorias de enfermagem desenvolvidas em programas de pós-graduação *stricto sensu* em enfermagem no Brasil.

## MÉTODOS

### Aspectos éticos

A pesquisa não foi submetida à apreciação em Comitê de Ética em Pesquisa com Seres Humanos, haja vista que foi realizada com dados secundários e de domínio público. No entanto, preservaram-se os direitos autorais dos estudos.

### Desenho, período e local do estudo

Trata-se de estudo bibliométrico realizado a partir da análise das dissertações e teses oriundas dos programas de pós-graduação em enfermagem brasileiros depositados no Catálogo de Teses e Dissertações da CAPES. Optou-se por esse tipo de trabalho por compor o alto nível de produção científica no país, apresentando rigor metodológico nos estudos, validados em bancas de qualificação.

Os estudos foram localizados pela ferramenta de busca do Catálogo de Teses e Dissertações da CAPES, com uso de Descritores em Ciências da Saúde (DeCS): “Teoria de Enfermagem” e “Modelos de Enfermagem”. Também foram aplicados os termos “Teoria” e “Teoria de Médio Alcance”, sendo implementado o *Preferred Reporting Items for Systematic Reviews and Meta-Analyses* (PRISMA). Não houve delimitação de marco temporal. A coleta de dados ocorreu em outubro de 2023.

### Critérios de inclusão e exclusão

Foram incluídas dissertações e teses que desenvolveram teorias de enfermagem, sem recorte temporal. Foram excluídas as pesquisas que não estavam disponíveis online.

### Protocolo do estudo

A busca e a extração dos dados foram realizadas por duas pesquisadoras autoras do estudo. As divergências foram discutidas entre todos os autores.

Foram aplicadas as seguintes estratégias no campo de busca no Catálogo de Teses e Dissertações da CAPES: “Teoria de Enfermagem” (descritor) *OR* Teorias de Enfermagem” (palavra-chave), sendo encontrados 260 estudos; “Modelos de Enfermagem” (descritor) *OR* “Modelo de Enfermagem” (palavra-chave), sendo encontrados 31 estudos; “Teoria de Médio Alcance” (palavra-chave), sendo encontrados 49 estudos; e “Teoria” *OR* “Teorias” (palavras-chave), sendo encontrados 1.793 estudos. Foram utilizados os filtros “ciências da saúde” da “grande área conhecimento” e “enfermagem”/“enfermagem de saúde pública”/“enfermagem obstétrica”/“enfermagem pediátrica” da “área de conhecimento”. Após leitura dos títulos e resumos, 1.904 estudos foram excluídos por não abordarem desenvolvimento de teorias. A amostra final foi composta por 39 estudos (Figura 1).

**Figura 1:** Fluxograma de seleção dos estudos adaptado do PRISMA, Rio de Janeiro, Rio de Janeiro, Brasil, 2023

### Análise dos resultados e estatística

Os dados foram registrados em uma planilha eletrônica que incluía informações de ano de realização, título do estudo, palavras-chave, objetivo do estudo, universidade, autor, orientador, programa, nome da teoria/modelo desenvolvido, tipo de estudo, referencial teórico/metodológico e área de conhecimento estabelecidas pelos autores (saúde da mulher, saúde do adulto/idoso, saúde da criança/adolescente e saúde mental).

Os dados catalogados foram analisados pelos autores do estudo, que possuem doutorado e *expertise* em pesquisas sobre teorias de enfermagem, além de publicações acadêmicas nessa área.

Para análise dos dados, foi utilizada estatística descritiva com frequência absoluta e relativa, e os achados foram apresentados em quadros/tabelas.

## RESULTADOS

Foram encontradas 39 teorias de enfermagem produzidas e difundidas em 26 (66,7%) teses e 13 (33,3%) dissertações, durante o período de 2012 a 2023, em 13 programas de pós-graduação de três regiões geográficas do Brasil. O Quadro 1 apresenta uma síntese do perfil das teorias (T), organizado por ordem crescente de ano, informando o título da tese ou dissertação, instituição, autorias, orientadores e objetivos principais.

**Quadro 1:** Distribuição das teses e dissertações produzidas no Brasil acerca de desenvolvimento de teorias de enfermagem (n=39), Rio de Janeiro, Rio de Janeiro, Brasil, 2023

Nº ano universidade	Tese/dissertação Título	Autor Orientador	Principais objetivos
(T1) 2012 UFRJ	Tese: Proposição de uma Teoria de Enfermagem para o Processo de Interação em Ambientes Virtuais^(12)^	Autor: Jaqueline Santos de Andrade Martins Orientador: Marcos Antônio Gomes Brandão	Propor uma teoria de enfermagem para o processo de interação em ambientes virtuais a partir das estratégias de construção de teoria proposta por Walker e Avant.
(T2) 2015 UFRJ	Tese: Teoria de Médio Alcance de Amamentação: tecnologia de cuidado^(13)^	Autor: Cândida Caniçali Primo Orientador: Marcos Antônio Gomes Brandão	Elaborar uma TMA de amamentação com base no Modelo Conceitual de Sistemas Abertos de Imogene King.
(T3) 2016 UFRJ	Tese: Teoria de Médio Alcance de Enfermagem para Atenção à Saúde Mental^(14)^	Autor: Wilson Denadai Orientador: Marcos Antônio Gomes Brandão	Propor uma TMA de enfermagem para a saúde mental sustentado em Imogene King e nos aspectos conceituais e filosóficos do Sistema Único de Saúde.
(T4) 2017 UFC	Dissertação: Padrão respiratório ineficaz em crianças com cardiopatias congênitas: construção e validação por juízes de uma Teoria de Médio Alcance^(15)^	Autor: Nayara Maria Gomes de Souza Orientador: Viviane Martins da Silva	Verificar a validade da estrutura do diagnóstico de enfermagem (DE) Padrão Respiratório Ineficaz em crianças com cardiopatias congênitas pela realização de duas etapas, como construção de uma TMA e análise por juízes.
(T5) 2018 UECE	Dissertação: Reabilitação cardiovascular: proposta de uma teoria de enfermagem de médio alcance^(16)^	Autor: Maria Sinara Farias Orientador: Lucia de Fatima da Silva	Desenvolver uma TMA de enfermagem a partir de estratégias de desenvolvimento de teorias propostas por Walker e Avant para o processo de reabilitação cardiovascular.
(T6) 2018 UECE	Tese: Teoria de enfermagem de médio alcance para adesão de pessoas ao tratamento de hipertensão arterial e diabetes mellitus^(17)^	Autor: Kalidia Felipe de Lima Costa Orientador: Maria Vilani Cavalcante Guedes	Elaborar e validar uma TMA de enfermagem para adesão de pessoas ao tratamento de hipertensão arterial e diabetes mellitus.
(T7) 2018 UFRN	Tese: Construção e validação do diagnóstico de enfermagem risco de volume de líquidos excessivo a partir de uma Teoria de Médio Alcance^(18)^	Autor: Maria Isabel da Conceição Dias Fernandes Orientador: Ana Luísa Brandão de Carvalho Lira	Construir e validar o DE Risco de Volume de Líquidos Excessivo em pacientes submetidos à hemodiálise a partir de uma TMA.
(T8) 2019 UFSC	Dissertação: Modelo Teórico de Cuidado de Enfermagem às Mulheres em Tratamento de Câncer de Mama na Saúde Suplementar^(19)^	Autor: Ana Carolina Custódio Orientador: Evangelia Kotzias Atherino dos Santos	Desenvolver um modelo teórico de cuidado de enfermagem às mulheres em tratamento de câncer de mama à luz do pensamento complexo no setor da saúde suplementar.
(T9) 2019 UFSM	Tese: Estressores e variances de bem-estar em pessoas idosas hospitalizadas: Teoria de Médio Alcance de enfermagem^(20)^	Autor: Eliane Raquel Rieth Benetti Orientador: Margrid Beuter	Desenvolver uma TMA de enfermagem sobre estressores e variances de bem-estar em pessoas idosas hospitalizadas a partir da síntese teórica dos dados empíricos e da derivação de conceitos do Modelo de Sistemas de Neuman.
(T10) 2019 UFPB	Tese: Subconjunto terminológico da CIPE^®^ para pessoas com síndrome metabólica: base conceitual para a Teoria de Médio Alcance do cuidado no contexto de risco cardiovascular^(21)^	Autor: Nuno Damácio de Carvalho Félix Orientador: Maria Miriam Lima da Nóbrega	Analisar os conceitos risco cardiovascular e síndrome metabólica; estruturar um subconjunto terminológico da CIPE^®^; realizar a aplicação clínica do subconjunto; e desenvolver uma TMA para o cuidado no contexto de risco cardiovascular.
(T11) 2019 UFC	Tese: Validação do diagnóstico de enfermagem resposta disfuncional ao desmame ventilatório (RDDV)^(22)^	Autor: Larissa de Araújo Lemos Orientador: Marcos Venício de Oliveira Lopes	Estabelecer a validade do DE Resposta Disfuncional ao Desmame Ventilatório da NANDA-I e elaborar uma TMA sobre desmame ventilatório disfuncional.
(T12) 2019 UFPE	Dissertação: Validação do conteúdo do diagnóstico de enfermagem “conhecimento deficiente em indivíduos com insuficiência cardíaca”^(23)^	Autor: Claudia Gabrielle da Silva Orientador: Cecília Maria Farias de Queiroz Frazão	Validar o conteúdo do DE Conhecimento Deficiente em indivíduos com insuficiência cardíaca e construir uma TMA com a identificação dos atributos, antecedentes e consequentes.
(T13) 2020 UFRJ	Tese: Teoria do risco de padrão glicêmico desequilibrado em adultos e idosos com diabetes mellitus em tratamento^(24)^	Autor: Rafael Oliveira Pitta Lopes Orientador: Marcos Antônio Gomes Brandão	Desenvolver uma teoria sobre variações glicêmicas em adultos e idosos com diabetes mellitus em tratamento, tendo como base o Modelo Conceitual da Adaptação de Roy.
(T14) 2020 UFC	Tese: Teoria de situação específica e análise de conteúdo do diagnóstico de enfermagem nutrição desequilibrada: menor do que as necessidades corporais em crianças com câncer^(25)^	Autor: Iane Ximenes Teixeira Orientador: Marcos Venício de Oliveira Lopes	Desenvolver duas etapas do processo de validação de DE com o intuito de obter evidências de validade teórica e de conteúdo do DE Nutrição Desequilibrada: Menor do que as Necessidades Corporais no contexto de crianças com câncer.
(T15) 2020 UFRN	Tese: Ressecamento Ocular: desenvolvimento de uma teoria de enfermagem de médio alcance^(26)^	Autor: Ana Paula Nunes de Lima Fernandes Orientador: Allyne Fortes Vitor	Desenvolver uma TMA de enfermagem do ressecamento ocular com base no Modelo de Adaptação de Roy.
(T16) 2020 UFPE	Tese: Validação do diagnóstico de enfermagem rede social de apoio ineficaz^(27)^	Autor: Michelline Santos De Franca Orientador: Cleide Maria Pontes	Validar o DE Rede Social de Apoio Ineficaz e elaborar a TMA Ineficácia da Rede Social de Apoio.
(T17) 2020 UFC	Tese: Validação do diagnóstico de enfermagem proteção ineficaz em adolescentes com câncer^(28)^	Autor: Marilia Mendes Nunes Orientador: Marcos Venício de Oliveira Lopes	Estabelecer uma estrutura diagnóstica que represente evidência de validade para inferência do DE Proteção Ineficaz em adolescentes com câncer e construir uma TMA.
(T18) 2021 UFRN	Dissertação: Construção de uma teoria de médio alcance para o diagnóstico de enfermagem estilo de vida sedentário^(29)^	Autor: Renata Marinho Fernandes Orientador: Ana Luísa Brandão de Carvalho Lira	Construir uma TMA para o DE Estilo de Vida Sedentário em adolescentes e adultos jovens.
(T19) 2021 UFPR	Tese: Teoria de enfermagem de médio alcance para o cuidado transpessoal domiciliar^(30)^	Autor: Luana Tonin Orientador: Maria Ribeiro Lacerda	Construir uma TMA de enfermagem para o cuidado transpessoal domiciliar.
(T20) 2021 UFRN	Dissertação: Sobrepeso em adolescentes e adultos jovens: uma teoria de médio alcance^(31)^	Autor: Ana Carolina Costa Carino Orientador: Ana Luísa Brandão de Carvalho Lira	Construir uma TMA para o DE Sobrepeso em adolescentes e adultos jovens.
(T21) 2021 UFRN	Tese: Teoria de médio alcance para o cuidado de enfermagem ao adolescente com obesidade na atenção primária à saúde^(32)^	Autor: Anne Karoline Candido Araújo Orientador: Ana Luísa Brandão de Carvalho Lira	Desenvolver uma TMA para o cuidado de enfermagem na Atenção Primária à Saúde ao adolescente com obesidade.
(T22) 2021 UFSM	Tese: Cuidado centrado à pessoa idosa institucionalizada com demência: teoria de médio alcance de enfermagem^(33)^	Autor: Larissa Venturini Orientador: Margrid Beuter	Elaborar uma TMA para o cuidado de enfermagem à pessoa idosa institucionalizada com demência, sustentada na Abordagem Centrada na Pessoa de Carl Rogers.
(T23) 2021 UFMA	Dissertação: Análise de conteúdo do diagnóstico de enfermagem perfusão tissular periférica ineficaz em pacientes com pé diabético^(34)^	Autor: Lorrany Fontenele Moraes Da Silva Orientador: Livia Maia Pascoal	Analisar o conteúdo do DE Perfusão Tissular Periférica Ineficaz em pacientes com pé diabético a partir de uma TMA.
(T24) 2022 UFRN	Dissertação: Teoria de médio alcance do diagnóstico de enfermagem de risco de aspiração em pacientes críticos adultos^(35)^	Autor: Priscila Kaline de Andrade Silva Rocha Orientador: Ana Luísa Brandão de Carvalho Lira	Desenvolver uma TMA do DE Risco de Aspiração em pacientes críticos.
(T25) 2022 UFSM	Tese: Microteoria para cuidados de enfermagem na prevenção do delirium em pessoas idosas na unidade de terapia intensiva^(36)^	Autor: Sandra da Silva Kinalsk Orientador Margrid Beuter	Desenvolver uma microteoria, do tipo prescritiva, para cuidados de enfermagem na prevenção do *delirium* em pessoas idosas na Unidade de Terapia Intensiva, sustentada em construtos do Modelo de Adaptação de Callista Roy.
(T26) 2022 UFRN	Tese: Desenvolvimento de teoria de médio alcance de enfermagem para letramento em saúde prejudicado^(37)^	Autor: Amanda Barbosa da Silva Orientador: Allyne Fortes Vitor	Desenvolver uma TMA para o letramento em saúde prejudicado.
(T27) 2022 UFPB	Tese: Aplicabilidade clínica do subconjunto terminológico da CIPE^®^ para a mulher idosa com vulnerabilidade relacionada ao HIV/AIDS: desenvolvimento de uma teoria de médio alcance^(38)^	Autor: Marcia Cristina de Figueiredo Santos Orientador: Maria Miriam Lima da Nóbrega	Avaliar a aplicabilidade clínica do subconjunto terminológico da CIPE^®^ para a mulher idosa com vulnerabilidade relacionada ao HIV/aids, estruturando uma TMA.
(T28) 2022 UFRJ	Tese: Teoria de médio alcance do processo adaptativo da pessoa com lesão na medula espinhal baseado no modelo de adaptação de Roy^(39)^	Autor: Kenia Rocha Leite Zaccaro Orientador: Marcos Antônio Gomes Brandão	Construir uma TMA do processo adaptativo da pessoa com lesão traumática da medula espinhal baseada no Modelo de Adaptação de Callista Roy.
(T29) 2022 UFRGS	Tese: Teoria de médio alcance da dimensão existencial do ser no mundo da doença renal crônica^(40)^	Autor: Carolina Giordani da Silva Orientador: Maria da Graça Oliveira Crossetti	Desenvolver uma TMA da dimensão existencial do ser no mundo da doença renal crônica embasada na Teoria Humanística de Enfermagem.
(T30) 2022 UFF	Tese: Diagnóstico de enfermagem Letramento em Saúde Insuficiente na População Idosa: Estudo de validação^(41)^	Autor: Rachel da Silva Serejo Cardoso Orientador: Rosimere Ferreira Santana	Avaliar evidências de validade conceitual, de conteúdo e teórico-causal da proposta diagnóstica Letramento em Saúde Insuficiente para idosos. TMA explicativa a partir do DE Letramento em Saúde Insuficiente na pessoa idosa.
(T31) 2022 UFRN	Dissertação: Teoria de médio alcance para a Autogestão Ineficaz da Saúde no cuidado de pessoas vivendo com o Vírus da Imunodeficiência Humana^(42)^	Autor: Rebecca Stefany Da Costa Santos Orientador: Richardson Augusto Rosendo da Silva	Desenvolver uma TMA de enfermagem para a Autogestão Ineficaz de Saúde em pessoas vivendo com o Vírus da Imunodeficiência Humana à luz do Modelo de Adaptação de Roy.
(T32) 2022 UFC	Tese: Validação do diagnóstico de enfermagem baixa autoeficácia em saúde^(43)^	Autor: Reinaldo Gutierrez Barreiro Orientador: Marcos Venício de Oliveira Lopes	Validar o construto diagnóstico de Baixa Autoeficácia em Saúde para possível incorporação na Classificação de DE da NANDA *International* e elaborar uma TMA sobre a Baixa Autoeficácia em Saúde.
(T33) 2022 UFPE	Dissertação: Validade de Conteúdo do diagnóstico de enfermagem Enfrentamento Ineficaz para Pacientes Oncológicos^(44)^	Autor: Ingrid Andrade Lima Orientador: Eliane Maria Ribeiro de Vasconcelos	Investigar a validade de conteúdo do DE Enfrentamento Ineficaz para pacientes oncológicos, construído a partir de uma TMA.
(T34) 2022 UFC	Dissertação: Validação de conteúdo do diagnóstico de enfermagem “síndrome pós-trauma” no contexto da violência contra a mulher^(45)^	Autor: Yanka Alcantara Cavalcante Orientador: Marcos Venício de Oliveira Lopes	Verificar a validade estrutural do DE Síndrome Pós-Trauma (SPT), mediante a produção de duas etapas, como construção de uma teoria de situação específica (TSE), por meio das fases de uma TMA, e a validação de conteúdo por juízes.
(T35) 2023 UFRJ	Tese: Teoria do risco de quedas em pessoas idosas na comunidade^(46)^	Autor: Jéssica de Castro Santos Orientador: Marcos Antônio Gomes Brandão	Construir uma teoria de enfermagem sobre o risco de quedas em pessoas idosas na comunidade.
(T36) 2023 UFRN	Tese: Desenvolvimento e Validação de Conteúdo da Proposição do diagnóstico de enfermagem Estresse Ocupacional a partir de uma teoria de médio alcance^(47)^	Autor: Romanniny Hevillyn Silva Costa Almino Orientador: Richardson Augusto Rosendo da Silva	Desenvolver e validar o conteúdo da proposição do DE Estresse Ocupacional.
(T37) 2023 UFRN	Dissertação: Desenvolvimento de uma teoria de médio alcance de Enfermagem para a Tensão do papel de cuidado^(48)^	Autor: Barbara Ebilizarda Coutinho Orientador: Allyne Fortes Vitor	Desenvolver uma TMA de enfermagem para a Tensão do Papel de Cuidador.
(T38) 2023 UNIFESP	Tese: Teoria de situação-específica para o controle da saúde na insuficiência cardíaca^(49)^	Autor: Gisele Saraiva Bispo Hirano Orientador: Alba Lúcia Bottura L. de Barros	Construir uma TSE que defina e descreva o controle da saúde de pacientes ambulatoriais com insuficiência cardíaca.
(T39) 2023 UFRN	Dissertação: Desenvolvimento de teoria de médio alcance de enfermagem para comportamentos ineficazes de manutenção da saúde em pessoas com condições crônicas^(50)^	Autor: Ana Clara Dantas Orientador: Allyne Fortes Vitor	Desenvolver uma TMA de enfermagem para os Comportamentos Ineficazes de Manutenção da Saúde em pessoas com condições crônicas.

A Tabela 1 apresenta a região do Brasil onde se localiza o programa de pós-graduação dos autores da teoria e detalha as instituições de ensino superior.

**Tabela 1:** Caracterização das dissertações e teses segundo as regiões do Brasil e a instituição de ensino superior proveniente (n=39), Rio de Janeiro, Rio de Janeiro, Brasil, 2023

Região	Instituição de ensino superior	Dissertação n	Tese n	Total f (%)
Nordeste	Universidade Federal do Rio Grande do Norte (UFRN)	6	5	11(28,2)
	Universidade Estadual do Ceará (UECE)	1	1	2 (5,1)
	Universidade Federal do Ceará (UFC)	2	4	6 (15,4)
	Universidade Federal da Paraíba (UFPB)	0	2	2 (5,1)
	Universidade Federal do Pernambuco (UFPE)	2	1	3 (7,7)
	Universidade Federal do Maranhão (UFMA)	1	0	1 (2,6)
Sudeste	Universidade Federal do Rio de Janeiro (UFRJ)	0	6	6 (15,4)
	Universidade Federal de São Paulo (UNIFESP)	0	1	1 (2,6)
	Universidade Federal Fluminense (UFF)	0	1	1 (2,6)
Sul	Universidade Federal de Santa Catarina (UFSC)	1	0	1 (2,6)
	Universidade Federal de Santa Maria (UFSM)	0	3	3 (7,7)
	Universidade Federal do Paraná (UFPR)	0	1	1 (2,6)
	Universidade Federal do Rio de Janeiro (UFRGS)	0	1	1 (2,6)

Predominaram teorias produzidas em programas de pós-graduação da região Nordeste (64,1%), seguidas do Sudeste (20,5%) e Sul (15,4%). As 39 teorias (T) foram elaboradas por autores únicos e orientadas por 18 pesquisadores. O maior número de orientações de um mesmo orientador foi seis (T1, T2, T3, T13, T28, T35) da UFRJ, seguido por cinco (T7, T18, T20, T21 e T24) da UFRN e por cinco (T11, T14, T17, T32 e T34) da UFC. Esses orientadores representam 41,0% das orientações.

A predominância dos objetivos das pesquisas foi o de criar/desenvolver teorias de enfermagem para validação de DEs, seguida das teorizações clínicas de condições de interesse da enfermagem, inclusive para a proposição de ações prescritivas ou conexões com demais elementos da prática, como resultados e intervenções de enfermagem. A Figura 2 estabelece a evolução temporal entre as teorias geradas/criadas de DEs e as demais.

**Figura 2:** Distribuição evolutiva das teorias com foco em diagnósticos de enfermagem e outras condições ou conceitos centrais (n=39), Rio de Janeiro, Rio de Janeiro, Brasil, 2023

A maioria das teorias tem foco em conceitos diagnósticos, principalmente da classificação da NANDA *International* (NANDA-I), sendo uma tendência crescente ao longo dos anos a partir de 2020.

Em relação a grupos humanos ou populações-alvo, as teorias incluem adultos/idosos (71,8%; n=28), crianças/adolescentes (12,8%; n=5), mulheres (10,3%; n=4) e pessoas com transtornos em saúde mental (2,6%; n=1). Apenas uma teoria (2,6%; n=1), a primeira identificada (T1), não tinha relação direta com condições clínicas, sendo focada no processo de interação em ambientes virtuais.

Os autores classificaram suas teorias nas tipologias de médio e micro alcances, e TSE, empregando nove métodos/estratégias de teorização, como detalhado no Quadro 2.

**Quadro 2:** Descrição dos métodos/estratégias de teorização relacionados ao tipo de teoria desenvolvida (n=39), Rio de Janeiro, Rio de Janeiro, Brasil, 2023

Principais métodos/estratégias de teorização utilizadas	TMA	TSE	TMc	NI
Análise, síntese e derivação de conceitos, afirmações e teorias^(51)^.	T1, T2, T6, T9, T10, T15, T19, T21, T22, T27	T38		
Validade teórico-causal^(52)^.	T4, T5, T7, T11, T12, T16, T17, T18, T20, T23, T24, T26, T30, T31, T32, T33, T37, T39	T14, T34		
Uso de modelo conceitual explícito para guiar a pesquisa^(53)^.	T36			
Teoria Fundamentada nos Dados^(54)^.	T29			T8
Modelo de construção de teorias de Dubin^(55)^.				T13
Análise de modelo conceitual, desempacotamento do conhecimento e combinação teórica^(56-57)^.	T3			
Método geral de elaboração de teorias em disciplinas aplicadas^(58)^.				T35
Estratégia integrada classificação-teoria^(2)^.	T28			
Substrução teórica^(59-60)^.			T25	

*TMA - teoria de médio alcance, TSE - teoria de situação específica; TMc - teoria de micro alcance ou microteoria; NI - não informado.*

A maioria incluiu TMA (82,1%; n=32), seguida de TSE (7,7%; n=3), teoria de micro alcance ou microteoria (TMc) (2,6%; n=1), e em 7,7% (n=3) das pesquisas os autores não classificaram a teoria.

No que corresponde às estratégias mais prevalentes para desenvolvimento das teorias, 51,3% utilizaram validade teórico-causal de Lopes, Silva e Herdman; 28,2% utilizaram estratégias (análise, síntese e/ou derivação de conceitos, afirmações e teorias) de Walker e Avant; e 5,1% utilizaram a Teoria Fundamentada nos Dados (TFD).

## DISCUSSÃO

O mapeamento realizado traz evidências de um crescente movimento de desenvolvimento de teorias de enfermagem por mestrandos e doutorandos no Brasil, principalmente a partir de 2018, após os quatro^(12-15)^ estudos pioneiros verificados de 2012 a 2017. Tal achado contraria a ideia de uma degeneração no programa de pesquisa de teorias de enfermagem no país, particularmente para TMA de enfermagem.

É complexo dimensionar a amplitude do uso de teorias de enfermagem como referencial da prática profissional ou nas pesquisas clínicas de enfermagem, visto que exige sempre a análise de centenas de pesquisas que nem sempre explicitam claramente os referenciais teóricos. Por outro lado, é mais fácil mapear a construção teórica como empreendimento de pesquisa. Tais teorias são basicamente criadas para constituir fundamentação para a validade de DEs para a descrição, explicação ou predição de fenômenos e de condições de interesse para a prática clínica ou para guiar de forma prescritiva a prática profissional.

Também verificamos que o movimento iniciado no sudeste do Brasil se espalhou progressivamente para o Nordeste e depois para o Sul do país. Esse espraiamento tem relevância para a expansão da teorização, com a possibilidade de ampliação da capilarização e incorporação de traços culturais e regionais de um país de dimensões continentais.

A predominância de teorias desenvolvidas na região Nordeste, acelerada a partir de 2020, está associada à relevante contribuição dessa região do país no desenvolvimento de pesquisas em validação teórico-causal de DEs. Os atuais critérios para os níveis de evidência dos DEs da NANDA-I preveem o desenvolvimento de uma fundamentação teórica no nível de validade teórico-causal^(52,61)^. Também pode ter relação com esse movimento o crescimento dos programas de pós-graduação do Brasil, incentivados pelo Plano Nacional de Pós-Graduação da CAPES (PNPG 2011-2020)^(62)^.

Podemos interpretar como um esforço de teorização para a prática clínica de enfermagem o fato de 38 teorias de enfermagem abordarem diagnósticos ou condições de grupos ou populações humanas. Seja para grupos de adultos, idosos, crianças, mulheres ou grupos com agravos específicos, as teorias comunicam um direcionamento para a instrumentação de condições particulares do cuidado de enfermagem, afastando-se de temas ou construtos mais gerais, como das grandes teorias de enfermagem. Também indica menor interesse por teorização das dimensões de ensino ou gestão da prática profissional^(9)^.

O foco limitado também parece ter relação com as tipologias predominantes de TMA, TMc e TSE. Tais achados reforçam a perspectiva de níveis de pensamento teórico, em que as de médio alcance teriam escopo e abstração entre o específico para guiar a prática e pesquisa, contudo sem perder a capacidade de atender diferentes populações com o mesmo fenômeno.

A predominância das TMAs, verificada no mapeamento, converge para a tendência de teorização mundial na enfermagem, que se observou a partir das considerações do filósofo Frederick Suppe sobre a estrutura das teorias científicas e possivelmente de sua apresentação “*Middle range theories: what they are and why nursing science needs them*”, realizada, em 1993, no Simpósio do Conselho de Pesquisadores de Enfermagem da Associação Americana de Enfermeiros (*Council of Nurse Researchers Symposium* (ANA))^(63)^. Desde então, existe um contínuo interesse na construção de TMAs para esclarecer, descrever, prescrever e predizer os fenômenos, cenários, fatos, situações e intervenções que fazem parte do cotidiano da prática de enfermagem, contribuindo para o avanço da pesquisa e ciência em enfermagem^(64)^.

As TSEs foram a segunda tipologia mais verificada no mapeamento, e são também de baixo nível de abstração. Em seu formato original, seguiram traços mais práticos, contextualizados e culturais compatíveis com a vocação da Escola de Enfermagem da Universidade da Califórnia em São Francisco (*University of California at San Francisco School of Nursing*)^(6)^. Entre as raízes filosóficas que impulsionam as TSEs, encontra-se o pós-empirismo, que visa prever as experiências e respostas de um determinado grupo de seres humanos sob certas condições de saúde e doença^(65)^. Contudo, atualmente, tanto as estratégias de criação das TSEs quanto os fundamentos filosóficos são múltiplos^(65)^.

Nos níveis mais baixos de pensamento teórico, as TMcs são as menos formais, restritas em tempo, escopo e aplicação, compreendendo grupos de hipóteses de trabalhos ou proposições^(63)^. Esta pesquisa identificou o uso da substrução teórica como estratégia para criação da única microteoria^(36)^ identificada.

Por fim, o terceiro tipo de teoria de nível mais baixo de abstração, que são as teorias práticas, não foi encontrada nesta revisão. Teorias práticas surgiram na década de 1960 a partir das considerações de Dickoff *et al*.^(66)^ com abordagem de teorização orientada para a prática, possuindo níveis de isolamento de fatores, relacionamento de fatores, relacionamento de situações e produção de situações. Tais teorias teriam a meta final de modificar a realidade pela prescrição de situações, o que dependia das etapas preliminares de isolamento de conceitos com descrição, relacionamento de conceitos e afirmativas de natureza preditiva^(6)^.

Contudo, apenas recentemente, com o desenvolvimento de taxonomias e outros tipos de teorias, as teorias de prática puderam avançar para os desafios contemporâneos, pelo uso de métodos como a estratégia integrada classificação-teoria, por exemplo^(2)^.

O predomínio das estratégias de síntese, derivação e análise de conceitos, afirmações e teorias foi verificado na elaboração das teorias não diagnósticas de enfermagem. Para as teorias de Des, predominou a estratégia de validade teórico-causal.

Na estratégia de síntese, as informações baseadas na observação são usadas para construir um novo conceito, uma nova afirmação e uma nova teoria. A derivação consiste na transposição ou redefinição de um conceito, afirmação e teoria de um contexto ou campo para outro. Enquanto isso, na análise, o teórico deve dissecar o todo em partes para que possa ser melhor compreendido. Cada uma dessas estratégias pode ser usada para elaboração de conceitos, afirmações e/ou teorias^(51)^.

A estratégia de validade teórico-causal^(52)^ impulsionou a fundamentação teórica para a validação de DEs a partir de 2017. Tal estratégia, que é baseada no modelo proposto por Roy^(6)^, propõe desenvolvimento teórico partindo das seguintes etapas: definição da abordagem de construção da TMA; definição dos modelos teórico-conceituais a serem analisados; definição dos conceitos principais da TMA; desenvolvimento de um esquema pictorial; construção das proposições da TMA; estabelecimento das relações de causalidade e de evidências para a prática^(52)^.

Estratégias de teorização pouco consagradas no campo da enfermagem foram mais utilizadas por teoristas das regiões Sudeste e Sul do Brasil (T3, T8, T13, T25, T28, T29 e T35), indicando tendência para o emprego de novos métodos.

Podemos afirmar, pelos objetivos das pesquisas e a partir dos elementos produzidos na teorização, que os teoristas se ocuparam de um caráter de translação do conhecimento, pois, em sua maioria, propuseram algum elemento de natureza mais operacional, como diagramas, subconjuntos terminológicos, guias de ação/intervenção, links entre diagnósticos, resultados e intervenções, entre outros. Embora estivessem presentes os clássicos elementos da construção teórica, como conceitos, premissas, afirmativas, proposições e modelos icônicos, os autores produziram predominantemente elementos explicativos, preditivos ou prescritivos em suas teorias, o que traz uma interessante abordagem do quanto essas teorias poderiam contribuir com os propósitos das teorias práticas e da NTGP.

Esse aspecto das teorias aproximadas às condições da prática produz um contraponto para crítica da relevância das teorias de enfermagem para a profissão da enfermagem. Talvez a essência dessa crítica tenha base na percepção de alguns clínicos sobre algumas das grandes teorias de enfermagem norte-americanas. A lacuna teoria-prática tem sido descrita nos Estados Unidos da América entre 1980 e 1990, englobando diferenças entre as teorias ensinadas em sala de aula e a prática encontrada nas áreas de atuação clínica ou até mesmo na existência de uma desconexão entre as realidades teóricas e práticas^(67)^.

Assim, a progressão das pesquisas teóricas verificadas no Brasil pode ser um fator que evite ou minimize a degeneração do programa NTGP no Brasil, por oferecer novos referenciais que guiem as pesquisas e a prática clínica. As teorias mapeadas possuem um escopo para condições da prática clínica ou do preparo para o seu exercício em cenário desafiador (ambientes virtuais - T1).

Além disso, no conteúdo dessas teorias, podem estar contidos os aspectos regionais e as características do sistema de saúde do país. De fato, há que se ter um alinhamento entre teorias de enfermagem com a prática de profissionais para o alcance de boas e melhores práticas, especialmente para colaborar na solução dos desafios do Sistema Único de Saúde no Brasil^(68)^.

### Limitações do estudo

O estudo possui algumas limitações. O acesso na íntegra às teses e dissertações dependia de sua localização no portal da CAPES ou em repositórios institucionais. Assim, as teorias ainda não depositadas ou que não estavam em formato digital podem não ter sido incluídas no mapeamento. Para mitigar essas limitações, foi realizada uma busca livre na internet dos títulos das dissertações e teses selecionadas.

Outra limitação foi a utilização de um conjunto limitado de descritores no portal. Apesar de termos aplicado descritores controlados e termos não controlados relevantes, essa seleção restrita pode acarretar a perda de estudos pertinentes, como os relacionados às teorias produzidas pelo método da TFD. Vale ressaltar que, embora a TFD seja produtora de teorias, sua acepção de teorias substantivas e formais usualmente não é considerada pelos pesquisadores deste campo como alinhada à ideia mais difundida de teorias de enfermagem. Diante desse debate pouco avançado, optou-se por considerar apenas as teorias nas quais os autores claramente anunciaram uma perspectiva convergente para a visão de teorias de enfermagem adotada neste estudo.

### Contribuições para a área da saúde e da enfermagem

A investigação apresentou olhares para a produção científica de teorias de enfermagem brasileiras, destacando a necessidade de valorização e desenvolvimento de outros estudos, como os de validação e aplicação dessas teorias.

O mapeamento possibilitou uma compreensão da diversidade e amplitude das teorias de enfermagem desenvolvidas em contextos acadêmicos brasileiros, oferecendo uma visão do panorama atual do conhecimento na área. Além disso, forneceu *insights* valiosos para melhorar os programas de pós-graduação em enfermagem, permitindo que as instituições acadêmicas ajustem seus currículos e enfoques de pesquisa de acordo com as necessidades e tendências identificadas. O estudo também ajudou a identificar áreas em que as teorias de enfermagem ainda não foram amplamente exploradas ou desenvolvidas, destacando potenciais lacunas no conhecimento que podem ser alvos de futuras pesquisas.

## CONCLUSÕES

O estudo bibliométrico possibilitou conhecer as teorias de enfermagem desenvolvidas nos programas de pós-graduação no Brasil, o que representa um avanço na produção desse conhecimento na evolução profissional e na adoção de um programa de prática baseada em teorias.

É notório o avanço da enfermagem ao longo dos anos, observando-se o aumento de produções de teses e dissertações com pesquisas de desenvolvimento de teorias de enfermagem. As TMAs foram as mais prevalentes nessa bibliometria e estão em contínuo crescimento, porém com produções ainda limitadas no detalhamento teórico-metodológico de sua construção, o que dificulta a consulta das teses e dissertações como material educativo para desenvolvimento de teorias. No entanto, a utilização dessas produções científicas no país ainda é incipiente, considerando que as teorias dão a sustentação da ciência da enfermagem. Desse modo, é preciso a divulgação das teorias de enfermagem brasileiras para que sejam incorporadas na prática assistencial (como suporte na implementação do processo de enfermagem), no ensino e nas pesquisas.
