# Supplementary material for: ERRATUM
Source: Rev Bras Enferm. 2025 Jan 13;77(6):e2024n6e08. doi: 10.1590/0034-7167.20247706e08 (PMC11731846; doi:10.1590/0034-7167.20247706e08)
Supplement: Related texts

# Correction: ERRATA

**DOI:** 10.1590/0034-7167.20247706e08pt

No artigo “Teorias de enfermagem brasileiras de situação específica, médio e micro alcances: estudo bibliométrico”, com número DOI: https://doi.org/10.1590/0034-7167-2023-0520pt, publicado no periódico Revista Brasileira de Enfermagem, 2024;77(4):e20230520, Quadro 1:

Onde se lia:

Autor: Ingrid Andrade Lima

Orientador: Eliane Maria Ribeiro de Vasconcelos

Lê-se:

Autor: Ingrid Andrade Lima

Orientador: Jaqueline Galdino Albuquerque Perrelli
